# No Way, That’s Gross! How Public Exposure Therapy Can Overcome Disgust Preventing Consumer Adoption of Sustainable Food Alternatives

**DOI:** 10.3390/foods10061380

**Published:** 2021-06-15

**Authors:** Jan Andre Koch, Jan Willem Bolderdijk, Koert van Ittersum

**Affiliations:** Department of Marketing, University of Groningen, Nettelbosje 2, 9747 AE Groningen, The Netherlands; j.w.bolderdijk@rug.nl (J.W.B.); k.van.ittersum@rug.nl (K.v.I.)

**Keywords:** deviance, disgust, consumer behavior change, sustainable food alternatives

## Abstract

Two prominently discussed sustainable food alternatives—lab-meat and edible insects—elicit disgust among consumers, thereby preventing acceptance. While providing prospective consumers with more information on, for instance, the environmental benefits of lab-meat has shown some success in increasing consumer acceptance, we argue that the disgust response—the main barrier to the societal acceptance of these foods—is not addressed. This is, we argue, because disgust is not the result of misperceptions (e.g., edible insects carry diseases) and thus unlikely to be overcome by information alone. Building on the latest insights into the social origins of disgust, this manuscript reviews an alternative strategy to foster the broader acceptance of sustainable food alternatives that currently elicit disgust. Specifically, we explain why and how public exposure could be a promising avenue for marketers to reduce consumers’ disgust response and thus increase the acceptance of sustainable food alternatives.

## 1. Introduction

Greenhouse gas emissions and the entailing climate change are arguably one of the biggest threats to mankind’s prosperity in the 21st century. If the amount of greenhouse gases emitted into the Earth’s atmosphere will not be reduced significantly, the planet’s temperature will continue to rise and soon the world will reach a point of no return [1]. In response to this pending doomsday scenario, the Paris Agreement has been formulated, committing 196 countries to limit global warming [2]. In it, several domains of human behavior that need to change are addressed to ensure future prosperity for mankind [2].

Being the largest single contributor to human greenhouse gas emissions [3] and threatening biodiversity [4], the livestock sector has been the target of numerous proposed changes. As the livestock sector is a key provider of protein, alternative sources of protein have been reviewed, ranging from vegetarian options such as legumes to alternative sources of animal protein. Lab-meat and edible insects are two of the most prominent examples of alternative sources of animal protein. Lab-meat—meat produced by growing stem-cells in a laboratory—has been introduced as the closest potential alternative to conventional livestock [5]. Although assessments of the exact benefits of producing lab-meat over conventional livestock remain ambiguous [6], some suggest lab-meat may reduce greenhouse-gas emissions by a minimum of 78% while simultaneously significantly reducing the use of energy, land, and water [7]. Edible insects, similarly, would require less land, emit fewer greenhouse gases, require less feed, and be less susceptible to diseases transmittable to humans than conventional livestock [8,9]. 

Despite these promising benefits, their adoption has not yet occurred on the larger scale. Instead, lab-meat and edible insects receive the lowest acceptance of any sustainable food alternatives in Western societies [10]. Importantly, both lab meat [11] and edible insects [12,13] have been found to elicit disgust within consumers, a potent emotion that poses a significant challenge to their broader societal acceptance. Prospective consumers, for instance, have argued lab-meat to be “unnatural” [14] (p. 1)—a perception that has been found to drive the disgust response [10]. Similarly, edible insects, being linked to dirt [15] and infectious diseases [16], typically elicit disgust in Western consumers [12]. 

Targeting these self-reported concerns (e.g., providing factual information to correct the misperception that “edible insects carry diseases”), we argue, will not suffice to convert consumers, as disgust is difficult to reason with. Rozin, Millman, and Nemeroff [17], for instance, showed that participants still believed that a sterilized cockroach contaminated a drink, despite the fact that a sterilized cockroach—by definition—is sterile and thus free of contaminants. The mere idea of consuming something that had been in touch with a cockroach elicited disgust among participants, irrespective of whether the cockroach posed an actual threat. Thus, instead of trying to correct the misperception that lab-meat is unnatural [11] and unhealthy [18], and that edible insects carry diseases [15], we discuss an alternative route to reduce disgust that builds on the social origin of disgust. Whereas correcting misperceptions with information may fail to change consumers’ reaction toward sustainable food alternatives, we argue that increasing consumers’ exposure to the consumption of sustainable food alternatives has a better chance to reduce the consumers’ disgust response, and thereby making them more open to these alternatives. 

## 2. Why Do People Experience Disgust?

In order to help overcome consumers’ disgust and increase the adoption likelihood of sustainable food alternatives, such as edible insects and lab-meat, one first needs to thoroughly understand the disgust response: Why do people experience disgust in the first place? 

Disgust is one of the basic human emotions [19] and was initially tasked with ensuring the host’s well-being by preventing potential contaminants from entering the body [20]. This primal function of disgust thus mainly governed the ingestion of food that could pose a threat to the host (e.g., a rotten fruit) [19]. The typical disgust face—a gaping mouth, a wrinkled nose, and a raised upper lip—can be seen as an artefact of this core function of physically rejecting food [21]. Once triggered, disgust acts like a reflex, exhibiting a unified, singular response: behavioral avoidance [22]. 

Disgust dictates behavioral avoidance without much second-guessing [23] because falsely accepting a contaminant (e.g., eating a rotten fruit) would be much more costly than falsely rejecting a non-contaminant (e.g., forgoing what is mistaken to be a rotten fruit). Disgust thus acts according to the proverb “better safe than sorry”. 

Over time, disgust has evolved from an emotion mainly related to food, to become an “embodied loss aversion” [24] (p. 85), meaning that disgust is a physical reaction seeking to protect its host from impending loss of various origins, so going beyond the realm of foods. In contrast to fear, which also responds to threats [25], disgust now protects humans from more obscure and less imminent threats [26]. The specific elicitors of disgust are thus varied. Humans experience disgust over perceived carriers of diseases that may threaten one’s physical survival (e.g., others’ bodily fluids), over unfit sexual partners (e.g., incest), but humans can also be disgusted by immoral or non-normative behaviors (e.g., cheating) [27].

### The Social Nature of Disgust

Social norms reflect society’s implicit rules of social survival as they determine the most common or most acceptable behavior in any given situation [28]. Norms may describe common behavior, such as eating three meals a day, or they may prescribe proper behavior, such as being quiet in a library. A norm’s importance—displayed by the amount of compliance with it, the norm’s visibility, its enforcement, and the efforts in educating members of a society about this norm [29]—will affect whether people follow it. The more important a norm is perceived to be, the more likely people are to follow it. Importantly, the behavior of several individuals in the immediate social environment will influence what individuals perceive as the norm [30,31], and what they perceive to be proper behavior [32]. For instance, even if there were no signs explicitly indicating what the social etiquette is, any person would quickly perceive that being quiet is the correct thing to do when studying in a library: everybody is quiet and noisy people are shunned. 

Frequent exposure to a salient norm will facilitate its internalization in order to reduce the calculation costs of whether to adhere to the norm or risk social sanctions [29]. Once internalized, adherence to the norm is automatic and autonomous; internalized norms become manifested as convictions of right behavior [33], and nonnormative behavior (e.g., cheating) elicits disgust [27]. Having internalized society’s rules regarding what is and is not to be considered food, consumers are prone to experience disgust toward foods they perceive to be deviant irrespective of the specific qualities of the foods [34]. For instance, the consumption of dogs is supported in South Korea whereas it is an unthinkable and disgusting concept for most Western consumers [35]. 

In other words, whether a certain food elicits disgust or not partly depends on the social context. Given the social nature of disgust, we argue that changing the perception of deviance is key to increasing the acceptance of sustainable food alternatives. Once lab-meat, edible insects, or other sustainable food alternatives would no longer be considered socially deviant, but normal, disgust would cease, and these foods would simply be consumed just like any regular food. 

## 3. Public Exposure Therapy

The acceptance of foods within a society is constantly evolving. Foods once considered disgusting may become popular, as shown in the case of caviar, lobster, and sushi in Western society [36,37,38]. While the specific process accelerating the acceptance of these foods may differ, these examples show that foods that once were considered “disgusting” can become accepted or even highly sought after. Could sustainable food alternatives also elicit less disgust in the future?

We argue that because the disgust elicited by sustainable food alternatives is at least partly driven by the perception of deviance, increasing their visibility in the marketplace would, over time, dramatically change people’s reaction toward them. That is, because consumers have internalized society’s food norms and experience disgust at the thought of deviating from them [34]. This disgust response would become less intense once the food in question is considered to be less deviant.

Therefore, in order to effectively reduce disgust, we propose a public exposure therapy: consumers need to see these products in the marketplace and experiencing other people consuming these foods. This exposure would change the perceived norm, reduce the perception of deviance, and ultimately eliminate the disgust reaction over time. 

Exposure therapy, in therapeutic settings, gradually exposes the individual to the anxiety-inducing stimulus and thus showcases that the stimulus is harmless [39]. People suffering from arachnophobia—the fear of spiders—may, for example, be helped by exposure therapy, as it makes them experience first-hand that spiders pose no actual harm to them. Note that patients are not just informed that spiders are actually safe in order to convince them, but they experience it themselves through exposure. Similarly, instead of correcting common misperceptions with information (e.g., informing consumers that insects are fit for human consumption and that being created in a laboratory, lab-meat is as natural or unnatural as yoghurt) we argue it is more effective for consumers to experience sustainable food alternatives in the marketplace first-hand themselves. Treating disgust using exposure therapy allows people to reach a point where disgust is no longer elicited and thus does not affect behavior anymore—a goal that is sought after for sustainable food alternatives as well. 

**Figure 1:**
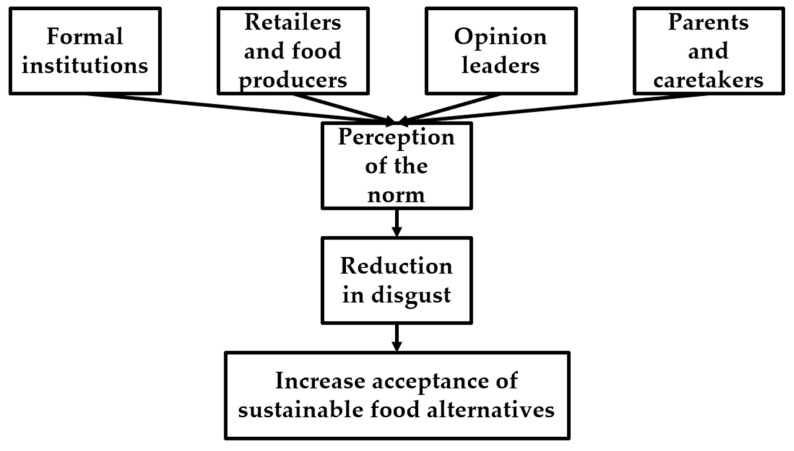
Public exposure therapy visualized. Different parties can affect the perception of the norm, thereby reducing disgust, and eventually increasing the acceptance of sustainable food alternatives.

We posit that targeting consumers’ internalized norms regarding food will change the disgusted consumers’ experience over sustainable food alternatives, such as lab-meat and edible insects. A change in consumers’ internalized norms may be initiated by exposing consumers to sustainable food alternatives and by treating these as normal foods in public settings—a sort of public exposure therapy. As we will discuss below, we propose that the salient behavior of, for instance, formal institutions, retailers and food producers, opinion leaders, and parents and caretakers could be an effective channel to normalize the consumption of sustainable food alternatives, and thus reduce the disgust response consumers would experience otherwise (Figure 1) [31,40,41]. 

### 3.1. Formal Institutions

Formal institutions, such as mass media, legal systems, educational institutions, and the government, are able to change people’s perception of what is normal through rules, policies, guidelines, and by simply displaying certain behaviors. People may interpret signals from such formal institutions to represent the majority’s opinion or, in other words, the norm [40]. For instance, the implementation of the tax on sugary drinks by the Mexican government in 2014 [42] or the requirement for all public canteens to offer a vegan meal option as brought into existence in Portugal in 2017 [43] may have been perceived by consumers as representing the opinion of the majority of people and thus signal a change in what most people approve of—the norm. Consumers may understand these policies to represent the will of the masses and thus perceive the majority of citizens to reject sugary drinks and welcome vegan food. Empirical evidence directly documenting the norm-shifting effects of formal institutions is scarce, yet Tankard and Paluck [44] find that a U.S. Supreme Court ruling supporting same-sex marriage changed the perception of U.S. citizens regarding how common and accepted same-sex marriage is. 

Similarly, institutional decisions, such as the recent approval of mealworms as a “novel food” by the European Commission [45], may change consumers’ perception of how deviant it is to eat insects, and thus over time help to reduce the disgust response. Specifically, the approval of insects as “novel food” may signal to consumers that the majority of people is in favor of eating insects and thus that compliance with the previously internalized norm that insects are not food is changing. Accordingly, as the norm that edible insects are not food would start to lose its importance due to the reduction in compliance, the norm may slowly be de-internalized and a new norm may emerge: insects are fit for humans as food. Likewise, if governments required all public canteens to offer at least one dish based on edible insects or lab-meat, the perception that consuming these foods is deviant would cease. 

In both these examples, the use of rulings and regulations could make consumers perceive the norm regarding sustainable food alternatives to differ from what they have internalized and thus slowly change it, thereby decreasing the intensity of the disgust consumers experience over the notion of eating insects or lab-meat. 

### 3.2. Retailers and Food Producers

Supermarkets, restaurants, food vendors, and food producers could further accelerate norm change with regard to sustainable food alternatives by, for example, producing, carrying, displaying, and labeling them saliently for consumers to see. 

A first step in making consumers aware that some sustainable food alternatives are already commonplace could, for instance, be to label foods that contain the EU- and FDA-approved [46,47], insect-based food colorant E120 more saliently. Via such labeling, consumers may realize that insects already have been a part of Western consumers’ diets for years, and thus consumers may understand that consuming insects is not as deviant as they thought. This labeling may familiarize people with the idea of eating insects and thus change the norm and reduce disgust. 

A similar strategy is explicitly pursued by producers of food containing insect flour who intentionally produce these foods with the goal to familiarize Western consumers with the concept of eating insects, and thus change the perceived norm that insects are not food in Western society [48]. Moreover, in a similar way as institutional decisions may signal the majority’s opinion, the voluntary behavior of retailers and other food producers (e.g., having insect-burgers on display) may signal to consumers that other consumers approve of these foods. Consumers may also infer retailers would only offer products for which sufficient demand exists; conversely, any food offered in a supermarket may be perceived as demanded and thus as normal. Following the same principle, supermarkets may also advertise sustainable food alternatives or provide recipes so that members of the society experience that the norm is changing. 

In doing so, the visibility of the norm that these sustainable food alternatives are not considered food could quickly change. Seeing others purchase these foods would—as a side-effect of retailers and food producers engaging with sustainable food alternatives—additionally signal that people’s compliance with this norm is deteriorating and, thus, also make consumers de-internalize the previous norm, thereby reducing the disgust response. 

### 3.3. Opinion Leaders

In addition to formal institutions, retailers, and food producers, we argue that highly influential individuals may change the perceived norm too, by engaging in a specific behavior and thus making this behavior more salient. Unless people find reasons not to, they generally tend to interpret the behavior of an individual member of a group to be representative of the entire group [30]. Accordingly, an influential person belonging to a specific group may challenge the perception of the norm within that group when engaging in deviant behavior. For instance, Bicchieri [49] discusses how a Peruvian TV show portraying the life of María, an illiterate single mother working her way up the social ladder by learning how to read, signaled a change in the perceived norm that adults do not seek further education. María’s behavior may have been interpreted as representative for working-class people and, by seeking adult education, María challenged and changed the viewers’ perception of what adults can and cannot do to improve their lives. If previously, viewers perceived adult education to be deviant behavior, watching the protagonist of a popular TV drama seeking adult education may have shown viewers that this was a misperception.

A similar effect could be imagined when celebrities or political figures engage in deviant behavior. If the president of the United States of America, for instance, would regularly and—this is crucial—publicly consume insect-based snacks or lab-meat, this behavior could be interpreted as being prototypically American and the norm that these sustainable food alternatives are not within what consumers consider food might quickly deteriorate as the norm’s visibility is reduced. The president’s behavior may influence people’s perception of what is normal and desirable behavior, thereby changing the amount of compliance with the norm and its visibility. 

A single, highly connected figure may thus change various aspects of the perceived norm’s importance, thereby challenging what people consider to be deviant, and eventually affect consumers’ disgust reaction to consuming sustainable food alternatives. Importantly, mere advocacy without displaying the target behavior is likely to be less influential, as actions typically speak louder than words when it comes to influencing norm perceptions [29]. 

### 3.4. Parents and Caretakers

What is perceived to be normal and thus the perception of deviance may also change organically over time, and does not always require top-down interventions. For instance, the parental age among U.S. citizens has been steadily increasing in recent decades [50,51], which means that older parents are becoming more and more common. This change may be due to increasing wealth, increasing longevity, a change in personal values, or a multitude of factors combined. Irrespective of the cause, the fact is that the norm is changing. Changes like this indicate that norms may change organically, but that this also may require a long timeframe. 

One way how the norms around sustainable food alternatives could organically change is if parents and other caretakers would decide to raise their children with the idea that eating insects or lab-meat is appropriate or even desirable. Except for an aversion to bitter taste, most of what children find disgusting is acquired through their social network and, as such, is malleable [52]. Feeding children edible insects and discussing that meat is created in a laboratory instead of by killing animals, these children likely will display acceptance toward edible insects and lab-grown meat as, to them, they are as normal as dinosaur-shaped chicken nuggets and colorful, sugary cereal. Growing up in this way, these children could internalize the norm that these food alternatives are acceptable and consequently would not express disgust at the thought of consuming them. 

However, for parents to raise their children to accept sustainable food alternatives, the parents themselves would have to accept them without displaying disgust, as parents transmit their feeling of disgust [53] and children tend to copy the eating behavior of their peers [54]. If parents no longer displayed a disgust response and the children perceived no deviance when consuming sustainable food alternatives, the children would not feel disgust toward these either. While this strategy might not change everyone’s behavior, the next generation would be raised during a period of transition and thus become more open-minded as the norm gradually deteriorates.

## 4. General Discussion and Future Research

Disgust is a major barrier to the adoption of two prominently discussed sustainable food alternatives—edible insets and lab-meat. In this manuscript, we reviewed a strategy that could help overcome disgust and thus accelerate the adoption of such foods. We propose that the social origin of disgust—deviance—can be tackled by public exposure therapy; via the salient behavior of formal institutions, food vendors, opinion leaders, and parents and caretakers, consumers could perceive widescale support for the consumption of sustainable food alternatives. This, in turn, would change consumers’ perception of these foods being deviant and consequently reduce their disgust reaction. 

Specifically, formal institutions, retailers and food producers, and opinion leaders are perceived to signal which behaviors the majority of society agrees upon and thus they can signal a change in what people perceive to be the norm [40]. Moreover, parents and caretakers have the capacity to change what those closest to them perceive to be normal. Parents, for instance, have a significant impact on what their children deem proper behavior, and thus what foods children deem to be disgusting. Additionally, marketers and policymakers could promote institutional acceptance of edible insects and lab-meat, seek opinion leaders to display the consumption thereof, and have food producers and vendors produce and distribute these foods to change what consumers perceive to be normal behavior. When consumers repeatedly see sustainable food alternatives in supermarkets and restaurants, see them being consumed by others without experiencing any negative repercussions, the mental image of the behavior being deviant will deteriorate, disgust will cease, and acceptance will inevitably follow. Thus, through public exposure therapy, the perception of the norm would change and disgust would gradually fade as the behavior becomes normal.

The individual strategies reviewed in this conceptual paper require additional research to test their effectiveness. For instance, the approval of mealworms as a “novel food” by the European Commission [45] may be as strong of an institutional signal as there is, as it affects millions of European consumers at once. Future research should track how the public opinion of mealworms as food changes following this categorization, and how effective such an institutional signal is in tackling internalized norms, and whether disgust dissipates accordingly. Further, the fact that these edible insects have been labeled as a “novel” food could arguably also hinder this process, as “novel” may be mistaken for “deviant” by consumers. Additional research should look into this potential pitfall of this categorization.

Next to institutional decisions, the effectiveness of retailers and food producers in changing the perceived norm regarding sustainable food alternatives could be explored by, for instance, experimental research. Including edible insects in restaurant menus, canteens, supermarkets, or cookbooks, we argue, could influence consumers’ perceptions of whether these are normal foods. Field experiments should be used to determine how effective such interventions are, but also to examine how quickly interventions show results. While edible insects are often compared to sushi, it took sushi decades to gain mainstream appeal in the Western world despite having a significant advantage of fitting into preexisting diets [55], implying that the adoption of edible insects may take even longer than that of sushi. 

Similarly, additional research could look into the characteristics of potential opinion leaders to portray the consumption of sustainable food alternatives. As mentioned above, people interpret the behavior of individual members of the group to be typical of the entire group, unless something indicates that they are not representative [30]. In the case of internalized norms, it is possible that mere deviation (e.g., consuming insects) may already be perceived as an indicator that someone is not a prototype of the group, and thus the behavior of that person may not affect the perception of the norm. Future empirical research should study to what extent someone can deviate from the group’s perceived norm without indicating that they are not a part of the group.

In sum, this paper has proposed public exposure therapy as a viable approach to increase the acceptance of sustainable food alternatives such as edible insects or lab-meat. With this, we hope to have offered a feasible alternative to the current, likely less effective strategy of addressing misperceptions regarding sustainable food alternatives.
